# Weight gain and psychiatric treatment: is there as role for green tea and conjugated linoleic acid?

**DOI:** 10.1186/1476-511X-6-14

**Published:** 2007-05-03

**Authors:** Martin A Katzman, Leslie Jacobs, Madalyn Marcus, Monica Vermani, Alan C Logan

**Affiliations:** 1 START Clinic for the Mood and Anxiety Disorders, Toronto, Canada; 2 Department of Psychiatry, University of Toronto, Toronto, Canada; 3 Integrative Care Centre of Toronto, Toronto, Canada

## Abstract

Dietary supplement use is widespread in developed nations. In particular, patients who utilize mental health services also report frequent consumption of dietary supplements, often in relation to management of adverse events and specifically weight gain. Weight gain induced by psychotropic medications can further compound psychological distress and negatively influence compliance. Here we report on four cases of social anxiety disorder treated with the atypical antipsychotic quetiapine. Self-administration of conjugated linoleic acid and green tea extract may have influenced objective anthropomorphic measurements; each patient had an unexpected decrease in total body fat mass, a decrease in body fat percentage and an increase in lean body mass. Since weight gain is a common and undesirable side-effect with psychiatric medications, our observation strongly suggests the need for controlled clinical trials using these agents.

## Background

The prevalence of dietary supplement use has increased steadily over the last decade. Recent investigations show that 73 percent of US adults consume at least one type of dietary supplement during the course of a year [1]. Similar patterns of increased self-prescription of dietary supplements have also been reported among adults in Canada and the United Kingdom [2,3]. Individuals with mental health disorders, including anxiety and depression, are frequent consumers of dietary supplements [4,5]. While the efficacy of dietary and herbal supplements in mental health care is a matter of debate, the potential to produce adverse reactions and drug interactions is certain. Given this reality, mental health care providers have been directed to become informed practitioners and query patients on the use of complementary therapies, and dietary supplements in particular [4,6].

In our outpatient clinic we have been inquiring on supplement use among those with mood disorders and anxiety for a number of years. In the interest of safety, we ask patients to disclose current dietary supplement use, and since many are taking, or will be initiating pharmacotherapy, we recommend discussion prior to patient's self-prescription with supplements.

One significant area of dietary supplement use which can impact a psychiatric practice is that pertaining to weight loss. Patients with obesity-related depression and those attempting to address or prevent antidepressant or antipsychotic-induced weight gain are often attracted by the marketing of weight loss supplements. A recent audit of commercially available weight loss supplements in 73 different metropolitan retail outlets revealed 402 different products, with over 4000 separate ingredients purported to promote weight loss [7]. The investigators point out that among these ingredients there is little in the way of human research in weight control to support any of these non-prescription ingredients. Green tea, or more specifically the green tea catechins, and conjugated linoleic acid are two weight loss ingredients with some, albeit limited, human data. Some studies have shown that green tea extract is associated with increased weight loss due to enhancement of thermogenesis, a change which is generally attributed to the catechin epigallocatechin-gallate (EGCG) [8]. For example, one human study shows that 270 mg of EGCG increased 24-hour thermogenesis among human volunteers by over 40 percent [9]. A recent hospital-based study in Japan used computed tomography to determine that 690 mg of catechins and 136 mg of EGCG resulted in the loss of more than 26 square cm of abdominal fat over the course of three months vs. placebo [10]. Although the results are equivocal, animal studies and some human research suggests that conjugated linoleic acid (CLA) can decrease body fat mass and body fat percentage while promoting lean body mass [11]. A recent double-blind, placebo-controlled study at the University of Toronto investigated a commercially available blend of EGCG and CLA (270 mg EGCG, 3400 mg CLA daily – abs+, Genuine Health Inc, Toronto, Canada) found that the combination resulted in a significant body weight loss, both as group total weight loss and mean body weight change vs. placebo [12].

The established potential of antidepressants and the emerging value of atypical antipsychotic medications in the mood and anxiety disorder are tempered by the well documented adverse effect of weight gain among users. In fact, the increases in body weight are not only significant, they can occur in a matter of weeks. For example, research shows that atypical antipsychotic medication can cause a ten pound weight gain in as little as eight weeks [13]. Other short-term studies also show a rapid weight gain in less than 10 weeks [14]. In our experience with the mood and anxiety we have found that the informed consent associated with antipsychotic and antidepressant prescription leads to an inevitable desire to initiate scientifically-unsupported weight loss supplements in select patients. This brief review reports on four cases where concerns related to weight gain associated with antidepressant and atypical antipsychotic prescription resulted in self-treatment with a commercially available green tea EGCG-CLA combination known as (abs+).

## Brief report

The current observation involved four individuals whom we identified as suffering with the mood and anxiety disorders who described concerns related to weight gain and who disclosed that they would be co-initiating use of the previously mentioned green tea EGCG-CLA combination (abs+) upon prescription of their medications. All patients are routinely questioned on the current or planned use of dietary/herbal supplements in our clinic. While the EGCG-CLA combination is widely available in Canadian retail outlets, it was not sold, promoted, nor endorsed in our clinic. In a high-volume mental health practice, four patients disclosed intent to use the EGCG-CLA combination, and consented to objective weight-related measurements of skinfold thickness, before, during and at the time of discontinuing the self-prescribed EGCG-CLA combination. These four patients were not taking any other weight loss supplements and none made any dramatic changes to diet or physical activity levels.

Four adults with primary generalized anxiety disorder were treated with the atypical antipsychotic quetiapine (Seoquel^®^) for periods ranging from 10–24 weeks. Patients reported co-self-administration of 270 mg EGCG, 3400 mg CLA daily in addition to the atypical antipsychotic medications and antidepressants they had taken for treatment resistant generalized anxiety disorders. Measurements of percent body fat were undertaken using the same method, first described initially by Durnin and Rahaman [15]. The process of measuring skin fat was undertaken using the Holtain skinfold caliper to measure subcutaneous adipose tissue. This process was repeated on three successive occasions at the same spot, by the assessor (MAK) three or more times until the assessment was of the subjects skin fat thickness. As well, all observations were made on the right side of the body. Assessments of skin thickness were undertaken at the chest, abdomen and thigh for men and the tricep, suprailliac spine and thigh according to the Jackson/Pollock 3 Caliper Method using the Jackson/Pollock equations [16]. The skin fold thickness was estimated in the three sites and entered into % body fat calculator using the Jakson/Pollock3 Caliper equation and accessible from Online Body Tracker [17]. Weight-related outcomes assessed in our clinic are described in tables 1, 2.

**Table 1:** Changes to total weight, body fat percentage (BF%), body fat mass (BFM) and lean body mass (LBM)

**Gender/Age**	**Quetiapine**	**Weeks**	**Total Weight**		**BF %**		**BFM**		**LBM**	
			
	**Dose/Day**	**on abs+**	**Start**	**End**	**Start**	**End**	**Start**	**End**	**Start**	**End**
Male; 44	600 mg	24	246.4	242	45.9	42.2	113.2	102.2	133.2	139.8
Female; 30	300 mg	14	176	176	38.1	35.2	67	62.2	109	113.8
Female; 35	200 mg	10	183	195.1	41.6	39.2	76.1	76.5	106.9	118.6
Male; 35	100 mg	20	217.1	227.1	41.5	39.4	90.1	89.5	127	137.6

**Table 2:** Percent changes to body fat percentage, body fat mass and lean body mass

**Gender/Age**	**% Change BFP**	**% Change BFM**	**% Change LBM**
Male; 44	-8.1	-9.7	4.9
Female; 30	-7.6	-7.2	4.4
Female; 35	-5.8	0.5	11
Male; 35	-5.1	-0.6	8.3

## Discussion

In these four adults who were taking quetiapine, the concurrent self-administration of green tea and conjugated linoleic acid appeared to be protective against gains in body fat. The results were fairly consistent; each had a decrease in total body fat mass, a decrease in body fat percentage and an increase in lean body mass. Interestingly, despite these desirable overall changes to body composition, there was an increase in total body weight in 2 patients. This may be, at least in part, due to the documented increases in lean body mass. Animal studies have consistently shown an increase in lean body mass and a reduction in body fat when CLA is incorporated into laboratory chow [18]. It also highlights a previous criticism of the weight-related endpoints used in studies on antidepressants and atypical antipsychotics; they have used varying assessments and have relied almost exclusively on total body weight and/or body mass index (BMI) [14]. Researchers have called for assessments that are more standardized and specific in nature, including the body fat and lean mass assessments used in our clinic.

The mechanisms whereby CLA and green tea EGCG can modulate energy balance remain mostly theoretical, although some researchers have provided insight. For example, CLA can inhibit prostaglandin E2, a chemical known to prevent lipolysis [18-21]. In addition, CLA may reduce lipid uptake by the fat cell, enhance thermogenesis in fat cells and increase the mobilization of fatty acids by an increase in catecholamine production [22].

Experimental studies show that green tea extract high in EGCG can directly inhibit gastric and pancreatic lipases, increase thermogenesis and possibly prevent the enzymatic degradation of catechol O-methyltransferase, an enzyme which plays a role in the respiration rate of brown adipose tissue, while animal research indicates that EGCG (not the related catechins) can significantly reduce food intake and body weight [8].

Obviously this report documents only the changes in body composition among just four adults on atypical antipsychotics. Clearly, there are external issues which may have changed outcome, including the evaluation of patients who were motivated to self-administer a commercial supplement. This, coupled with the awareness of our interest in any subsequent anthropomorphic changes, could serve to alter other weight-related lifestyle factors. Still, we find the consistency of the results among all of those followed to be remarkable and one which raises the possibility of a real effect. We set out to follow these patients with the expectation that there would be only increases in body fat mass and body fat percentages, with little to no change in lean body mass.

While no conclusions can be drawn, our observations should encourage a thorough scientific investigation concerning the utility of EGCG and CLA in the prevention of psychotropic medication-induced weight gain. Rapid weight gain among patients with mental disorders is an extremely distressing experience. In addition to the psychological fallout and potential non-compliance with otherwise effective medications, there is also the well known increase in risk of chronic diseases which accompany body fat accumulation. Every avenue of research should be exhausted in addressing side effects and medication-induced weight gain, including those related to the external products which mental health patients are self-prescribing.

Within the past five years author Alan C. Logan has been a compensated independent consultant for Genuine Health Inc, the company which manufactures abs+. He holds no stock or shares and possesses no financial holdings with Genuine Health. The product used is not patented or under active patent application. There are no other competing interests to report among any of the authors.

## Authors' contributions

MK and LJ evaluated the subjects and performed the anthropomorphic measurements, MM and MV conceived of the value of these observations, participated in subject identification, coordination and performed data analysis. AL acted as technical expert with properties of EGCG and CLA and drafted the manuscript. All authors reviewed the data, assisted in the final manuscript construction and agree to its contents.
